# BMC *Health Services Research* reviewer acknowledgement 2014

**DOI:** 10.1186/s12913-015-0764-9

**Published:** 2015-04-21

**Authors:** Christopher Morrey

**Affiliations:** BioMed Central, Floor 6, 236 Gray’s Inn Road, WC1X 8HB London, United Kingdom

**Perri 6**

UK

**Jos Aarts**

Netherlands

**Karina Aase**

Norway

**John Abebrese Boateng**

Ghana

**Seye Abimbola**

Nigeria

**Mauro Henrique Abreu**

Brazil

**Amani Abu- Shaheen**

Saudi Arabia

**Monica Acevedo**

Chile

**Ezekiel Taiwo Adebayo**

Nigeria

**Adedeji Adekanye**

Nigeria

**Marian Ådnanes**

Norway

**Martin Nyaaba Adokiya**

Ghana

**Willem Aelvoet**

Belgium

**Basema Afram**

Netherlands

**Bushra Afroze**

Pakistan

**Girdhar Agarwal**

India

**Sohail Agha**

USA

**Peter Agyei-Baffour**

Ghana

**Mohamed Azmi Ahmad Hassali**

Malaysia

**Maryam Ahmadian**

Malaysia

**Rukhsana Ahmed**

Indonesia

**Aima Ahonkhai**

USA

**Moses Aikins**

Ghana

**Bolajoko Ajoke Aina**

Nigeria

**James Akazili**

Ghana

**Halida Akhter**

Bangladesh

**Shahriar Akter**

Australia

**Patricia Akweongo**

Ghana

**Fadi Al Zoubi**

Canada

**Mohammed Alam**

UK

**Monira Alarouj**

Kuwait

**Tit Albreht**

Slovenia

**Vassilis Aletras**

Greece

**Yasmin Alhafaji**

Netherlands

**Syed Aljunid**

Malaysia

**Alejandro Allepuz**

Spain

**Sara Allin**

Canada

**Pascale Allotey**

Malaysia

**Helen Alm**

Sweden

**Carlos Alvarez**

USA

**Emmanuel Ameh**

Nigeria

**Joshua Amo-Adjei**

Ghana

**Seyi Ladele Amosun**

South Africa

**Eugenia Amporfu**

Ghana

**Elizabeth Andersen**

Canada

**Henning Boje Andersen**

Denmark

**Allen Anderson**

USA

**Kurt Anderson**

USA

**Philippa Anderson**

USA

**Ruth A. Anderson**

USA

**Vasanth Andrews**

UK

**Federica Angeli**

Netherlands

**Salvatore Annunziata**

Italy

**Rose Anorlu**

Nigeria

**Zahid Ansari**

Australia

**Fernando Antonanzas**

Spain

**Tony Antoniou**

Canada

**Jose Leopoldo Ferreira Antunes**

Brazil

**Konstantinos Antypas**

Norway

**Belkis Aracena**

Mexico

**Carlos Arango**

Colombia

**Edson Araujo**

USA

**Laura Arbour**

Canada

**Norm Archer**

Canada

**Greg Arling**

USA

**Cherie Armour**

UK

**Greg Armstrong**

Australia

**Joanna Armstrong Schellenberg**

UK

**Britt Arrelov**

Sweden

**Jelena Arsenijevic**

Netherlands

**Genevieve Aryeetey**

Ghana

**Augustine Asante**

Australia

**Raj Ashar**

USA

**Toni Ashton**

New Zealand

**Yaregal Asres**

Ethiopia

**Kostas Athanasakis**

Greece

**Vasilios Athyros**

Greece

**Roger Ayimbillah Atinga**

Ghana

**Lukoye Atwoli**

Kenya

**Carolyn Audet**

USA

**Asa Audulv**

Sweden

**Hanna Augustsson**

Sweden

**Isabelle Aujoulat**

Belgium

**Ahmed Awaisu**

Qatar

**Japheth Awiti**

Kenya

**Koku Awoonor-Williams**

Ghana

**Baffour Awuah**

Ghana

**Iben Axén**

Sweden

**Muhammad Ayub**

Pakistan

**Adriana Baban**

Romania

**Omar Badawi**

USA

**Franz G. Bader**

Germany

**Mario Bagat**

Croatia

**Cheick Oumar Bagayoko**

Mali

**Ilaria Baiardini**

Italy

**Frank Baiden**

Ghana

**Charles Bailey**

USA

**Daryl Bainbridge**

Canada

**Petra Baji**

Hungary

**Solome Kiribakka Bakeera**

Uganda

**Roland Bal**

Netherlands

**Eileen Baldry**

Australia

**Anne Balossier**

France

**Lawrence Barat**

USA

**Anna Maria Bargagli**

Italy

**Lawrence Barker**

USA

**Paul Barnett**

USA

**Mauro Barni**

Italy

**Hugh Barr**

UK

**Helen Barratt**

UK

**Patricia Barrionuevo**

Peru

**Melanie Barwick**

Canada

**Robert Basaza**

Uganda

**Caroline Bastiaenen**

Netherlands

**Lori Bastian**

USA

**Moses Bateganya**

Uganda

**Philip Batterham**

Australia

**Jennifer Baumbusch**

Canada

**Cynthia Baur**

USA

**Alia Bazzy-Asaad**

USA

**Davida Becker**

USA

**Katrien Beeckman**

Belgium

**Kendall Bein**

Australia

**Derek Bell**

UK

**Gill Bell**

UK

**Lorraine Bell**

Canada

**Mary Bell**

Canada

**Sandra Benavides**

USA

**Kathleen Beniuk**

Canada

**Paola Berchialla**

Italy

**Peter Berchtold**

Switzerland

**Sean Berenholtz**

USA

**Anne-Marie Bergh**

South Africa

**Werner Bergholz**

Germany

**Trine Strand Bergmo**

Norway

**Linda Bergofsky**

USA

**Julie Bernhardt**

Australia

**Allan Best**

Canada

**Kurt Bestehorn**

Germany

**Martie van Beuzekom**

Netherlands

**Ran Bhamra**

UK

**Upendra Bhojani**

India

**Rama Bhunia**

India

**Barbara Bien**

Poland

**Jean Joel Bigna**

Cameroon

**Ugur Bilge**

Turkey

**Stephen Billett**

Australia

**Aref Bin Abdulhak**

Saudi Arabia

**Sarah Birken**

USA

**Marie Bismark**

New Zealand

**Vesna Bjelic Radisic**

Austria

**Anne Mette Bjerkan**

Norway

**Wendy Bjornson**

USA

**Duane Blaauw**

South Africa

**Regis Blais**

Canada

**Christopher Blanchette**

USA

**Marc-André Blanchette**

Canada

**Jane Blazeby**

UK

**Ilse Blignault**

Australia

**Catherine Bliss**

USA

**Paul Blumenthal**

USA

**Jennifer Blumenthal-Barby**

USA

**Ian Blunt**

UK

**Thomas Bodenheimer**

USA

**Said Bodur**

Turkey

**J Ties Boerma**

Switzerland

**Peter Bogaty**

Canada

**Thomas Böhler**

Germany

**Philippe Boierling**

France

**Katrien Bombeke**

Belgium

**Gary Bond**

USA

**Cecile Boot**

Netherlands

**Josephine Borghi**

UK

**Sander Borgsteede**

Netherlands

**Mats Borjesson**

Sweden

**Ann Borzecki**

USA

**Thomas John Bossert**

USA

**Michel Botbol**

France

**Marge Bott**

USA

**Alex Bottle**

UK

**Michel Boudreaux**

USA

**Mary Boulton**

UK

**David Bowrey**

UK

**Laurent Boyer**

France

**Jennifer Boyko**

Canada

**Natalie Bradford**

Australia

**Elizabeth Bradley**

USA

**Sara Bradley**

USA

**Zoe Brady**

Australia

**Kate Brain**

UK

**Anneke Brand**

Netherlands

**Caroline Brand**

Australia

**Aase Brandt**

Denmark

**Susan Bratton**

USA

**Karen Bremner**

Canada

**Patricia Brennan**

USA

**Daniel Bressington**

UK

**Hans-Peter Brezinschek**

Austria

**Jackie Bridges**

UK

**William Brieger**

USA

**Annette Briley**

UK

**Michelle Bronze**

South Africa

**Peter Brooks**

Australia

**Edward Broughton**

USA

**George** Browman

Canada

**Jamie Brown**

UK

**Tony Brown**

USA

**Natasha Brusco**

Australia

**Maurice Bucagu**

Switzerland

**Angela Buchholz**

Germany

**Christoph Buhrer**

Germany

**Alexis Bujold**

Canada

**Yilmaz Bulbul**

Turkey

**Alicia Bunger**

USA

**Robert Burke**

USA

**Greer Burkholder**

USA

**Christopher Burton**

UK

**Gerard Bury**

Ireland

**Maria Antonella Burza**

Sweden

**Erik Buskens**

Netherlands

**Monica Busse**

UK

**Silvia Bustacchini**

Italy

**Sandra Butler**

USA

**David Byfield**

UK

**Jens Byskov**

Denmark

**Liam Caffery**

Australia

**Lucero Cahuana-Hurtado**

Mexico

**Stefano Calciolari**

Switzerland

**Amaia Calderón-Larrañaga**

Spain

**Jennifer Callaghan-Koru**

USA

**Claire Cameron**

New Zealand

**John Campbell**

Japan

**Carlos Campillo-Artero**

Spain

**Maureen Canavan**

USA

**Kenneth Candido**

USA

**Sandra Capra**

Australia

**Massimo Cardillo**

Italy

**Misericordia Carles**

Spain

**Siw Carlfjord**

Sweden

**Ken Carlson**

Algeria

**Eloise Carr**

Canada

**Henry Carretta**

USA

**Ewart Carson**

UK

**Nancy Carter**

Canada

**Thomas Cartier**

France

**Chantal Cases**

France

**Anne-Nicole Casey**

Australia

**Xavier Castells**

Spain

**Barbara Castelnuovo**

Uganda

**Víctor H Castillo**

Mexico

**Rodolfo De Almeida Lima Castro**

Brazil

**Enrique Castro-Sánchez**

UK

**Christa Cerra**

USA

**Merih Cetinkaya**

Turkey

**Chiyoung Cha**

South Korea

**Kalipso Chalkidou**

France

**Agnes Chan**

Taiwan

**Eric K H Chan**

Canada

**Jeremy Chancellor**

UK

**Rupa Chanda**

India

**Venkatraman Chandra-Mouli**

Switzerland

**Evelyn Chang**

USA

**Hsien-Yen Chang**

USA

**Jung Hyun Chang**

South Korea

**Jongwha Chang**

USA

**Yu-Chia Chang**

Taiwan

**Martin Charns**

USA

**Kath Checkland**

UK

**Zhuo Chen**

USA

**Saint S Chen**

Taiwan

**Gang Chen**

Australia

**Jie Chen**

USA

**Qi Chen**

USA

**Rong-Chi Chen**

Taiwan

**Wen Chen**

China

**Yingyao Chen**

China

**Wing-Sie Cheng**

Thailand

**Sudeh Cheraghi-Sohi**

UK

**Li-Nien Chien**

Taiwan

**Li-Yin Chien**

Taiwan

**Victor Chimhutu**

Norway

**Shang-Jyh Chiou**

Taiwan

**Jennifer Chipps**

Australia

**Karen Chiswell**

USA

**Anna Chiumento**

UK

**Eunice Chomi**

Tanzania

**Virasakdi Chongsuvivatwong**

Thailand

**Roger Chung**

Hong Kong

**Oriana Ciani**

UK

**Catia Cilloniz**

Spain

**Lauren Cipriano**

Canada

**Lauren Clack**

Switzerland

**Neree Claes**

Belgium

**Amanda Clare**

UK

**Alexander Clark**

Canada

**Jeffrey Clark**

USA

**Terryann Clark**

New Zealand

**David Clarke**

UK

**Philip Clarke**

Australia

**Robyn Clay-Williams**

Australia

**Kerri Clough-Gorr**

Switzerland

**Anne Cockcroft**

Botswana

**Hernan Cohen Arazi**

Argentina

**Raphael Cohen-Almagor**

UK

**Cynthia Colapinto**

Canada

**Tim Colbourn**

UK

**Anna Coleman**

UK

**Jamie Collins**

USA

**Mercedes Colomar**

Uruguay

**Tracy Comans**

Australia

**Elizabeth Comino**

Australia

**Anita Conforti**

Italy

**Richard Cook**

Sweden

**Evelyn Cornelissen**

Canada

**Tony Cornford**

UK

**Gloria Coronado**

USA

**Julieta Corral**

Spain

**Aline Corvol**

France

**Andrew Costa**

Canada

**Massimo Costantini**

Italy

**Fernando Coto**

Costa Rica

**Francesc Cots**

Spain

**Susan Cottrell**

UK

**Angela Coulter**

UK

**Simon Coulton**

UK

**Ian Couper**

South Africa

**Karen Courtney**

Canada

**Joseph Couto**

USA

**Janneke Cox**

Netherlands

**Peter Craig**

UK

**Peter Cram**

Canada

**Jane Murray Cramm**

Netherlands

**Grainne Crealey**

UK

**Andreea Creanga**

USA

**Torrey Creed**

USA

**Jo Crichton**

UK

**Bart Criel**

Belgium

**Clare Crowley**

UK

**Eduardo Cruz**

Portugal

**Joaquin Cubiella**

Spain

**Larry Culpepper**

USA

**Frances Cunningham**

Australia

**Maria Paula Curado**

France

**Alessandro Curto**

Italy

**Jonathan Cylus**

UK

**Carolyn Czoski Murray**

UK

**Matiwos Daba**

Ethiopia

**Katie Dainty**

Canada

**Gavin Daker-White**

UK

**David Dale**

USA

**Philip Ayizem Dalinjong**

Ghana

**Maurice Dalton**

USA

**Barbara Daly**

New Zealand

**Gianfranco Damiani**

Italy

**Laura Damschroder**

USA

**M Carolina Danovaro-Holliday**

USA

**Andriy Danyliv**

Ukraine

**Blair Darney**

USA

**Umakant Dash**

India

**Marie Dauvrin**

Belgium

**Larry Davidson**

USA

**Ruth Davies**

UK

**Keith Davis**

USA

**Peter Davis**

New Zealand

**Damon Daylamani-Zad**

UK

**Manuela De Allegri**

Germany

**Dinny De Bakker**

Netherlands

**Margaretha Christine De Bruijne**

Netherlands

**Ayesha De Costa**

Sweden

**Laure De Decker**

France

**Simon De Lusignan**

UK

**Victoria De Menil**

UK

**Matthieu De Stampa**

France

**Anniza De Villiers**

South Africa

**Linda De Villiers**

South Africa

**G. Ardine De Wit**

Netherlands

**Paquita De Zulueta**

UK

**Jonas Debesay**

Norway

**Anja Declercq**

Belgium

**Tom Decroo**

Belgium

**Ryan Deforge**

Canada

**Ellen Tveter Deilkås**

Norway

**Veronique Del Marmol**

Belgium

**Kim Delbaere**

Australia

**Cristina Del-Ben**

Brazil

**Derek Delia**

USA

**Thérèse Delvaux**

Benin

**Nadia Demarteau**

Belgium

**Petra Denig**

Netherlands

**Mike Dent**

UK

**Richard Derman**

USA

**Jane Desborough**

Australia

**Domino Determann**

Netherlands

**Mary Devany**

USA

**Angela Dew**

Australia

**Sanket Dhruva**

USA

**Paula Diab**

South Africa

**Vakaramoko Diaby**

USA

**Esperanza Diaz**

Norway

**Hassan H. Dib**

Canada

**Helen Dickinson**

Australia

**Virginia Dickson-Swift**

Australia

**Marjolein Dieleman**

Netherlands

**Hans Jochen Diesfeld**

Germany

**Vance Dietz**

USA

**Carmen Desiree Dirksen**

Netherlands

**Simon Dixon**

UK

**Mamuka Djibuti**

Georgia

**Fahdi Dkhimi**

Belgium

**Mai Do**

USA

**Charles Doarn**

USA

**Henry Doctor**

Nigeria

**Carmen Dolea**

Switzerland

**Sara Donetto**

UK

**Amol Dongre**

India

**Claire Donnellan**

Ireland

**Catherine Donnelly**

Canada

**Caroline Donovan**

Australia

**Diane Doran**

Canada

**William Doucette**

USA

**Lynda Doward**

UK

**Bryan Dowd**

USA

**Dawn Dowding**

USA

**Julia Downing**

Uganda

**Therese Dowswell**

UK

**Colleen Doyle**

Australia

**Aline Drapeau**

Canada

**David Dror**

India

**Arik Drucker**

Canada

**Renee Du Toit**

South Africa

**Stephen Duckett**

Canada

**Antoine Duclos**

France

**Dmitry Dukhovny**

USA

**James Dunbar**

Australia

**Cathy Duncan**

Australia

**Anna Dunér**

Sweden

**Alison Dunkley**

UK

**Luis Durán-Arenas**

Mexico

**Angela Durey**

Australia

**Jo Durham**

Australia

**Gilles Dussault**

Portugal

**Hemant Dwivedi**

India

**Rosamond Dwyer**

Australia

**Elin Dysvik**

Norway

**Mervyn Eadie**

Australia

**Phyllis Easton**

UK

**Jennifer E Edelman**

USA

**Dale Edgar**

Australia

**Dawn Edge**

UK

**Anbrasi Edward**

USA

**Alun Edwards**

Canada

**Jane Edwards**

Australia

**Louisa Edwards**

UK

**Judith Effken**

USA

**Mary Egan**

Canada

**Marlene J Egger**

USA

**Ashley Eggman**

USA

**Jan P Ehlers**

Germany

**Elizabeth Ekirapa-Kiracho**

Uganda

**Maguy El Hajj**

Qatar

**Ann Catrine Eldh**

Sweden

**Omar El-Gayar**

USA

**C Raina Elley**

New Zealand

**Martin Emmert**

Germany

**Liam Ennis**

UK

**David Epstein**

Spain

**Mirela Eric**

Serbia

**Erhan Eser**

Turkey

**Dominick Esposito**

USA

**Silvia Ess**

Switzerland

**Marie-Louise Essink-Bot**

Netherlands

**Carole A Estabrooks**

Canada

**Enyi Etiaba**

Nigeria

**Catrin Evans**

UK

**Roni Evans**

USA

**Combier Evelyne**

France

**Soludo Eze**

Nigeria

**Boniface Ikenna Eze**

Nigeria

**Echezona Ezeanolue**

USA

**Ogochukwu Ezeoke**

Nigeria

**Nkoli Ezumah**

Nigeria

**Esamai Fabian**

Kenya

**Reza Fadayevatan**

Iran

**Michael Falster**

Australia

**Victoria Fan**

USA

**Mahnaz Fanaian**

Australia

**Pengqian Fang**

China

**Yu Fang**

China

**Folayemi Faponle**

Nigeria

**Jane Farmer**

Australia

**Saeed Farooq**

UK

**Maryam Farooqui**

Malaysia

**Karen Farris**

USA

**Fereshteh Farzianpour**

Iran

**Denise Feil**

USA

**Bo Feng**

USA

**Zhanchun Feng**

China

**Jennifer Fenwick**

Australia

**Angeline Ferdinand**

Australia

**Laurie Anne Ferguson**

USA

**Laura Ferguson**

UK

**Ewan Ferlie**

UK

**Alfio Ferlito**

Italy

**Mazen Saleh Ferwana**

Saudi Arabia

**Bolanle Fetuga**

Nigeria

**Guido Filler**

Canada

**Sally Findley**

USA

**Sonja Firth**

Australia

**Belal Firwana**

USA

**Alastair Fischer**

UK

**Michael Fischer**

USA

**Ane Fisker**

Denmark

**Linda Fleisher**

USA

**Steffen Flessa**

Germany

**Monica Julie Fletcher**

UK

**Jan Florin**

Sweden

**Linda Fogarty**

USA

**Ted C T Fong**

Hong Kong

**Sharon Fonn**

South Africa

**Lindsay Forbes**

UK

**Jane Ford**

Australia

**Jay Ford**

USA

**Roberto Forero**

Australia

**Yvonne Forsell**

Sweden

**Louise Forsetlund**

Norway

**Martin Fortin**

Canada

**Kim Foster**

Australia

**Jean Christophe Fotso**

Kenya

**Deborah Fox**

Australia

**Gregory Fox**

Australia

**Margaret Fox**

UK

**Vassilis Fragoulakis**

Greece

**Anneke Leijntje Francke**

Netherlands

**Molly Franke**

USA

**Bryony Dean Franklin**

UK

**Randall Fransoo**

Canada

**Richard K Freeman**

USA

**Simon French**

Canada

**Atle Fretheim**

Norway

**Tobias Freund**

Germany

**Maria Freytsis**

USA

**Jan C Frich**

Norway

**Kevin Frick**

USA

**Roland Friele**

Netherlands

**Laura Fruhen**

UK

**Robert Fryatt**

USA

**Naomi Fulop**

UK

**Martha Funnell**

USA

**Kiyohide Fushimi**

Japan

**Anna Gagliardi**

Canada

**Arnaud Gagneur**

Canada

**Marie-Pierre Gagnon**

Canada

**Petros Galanis**

Greece

**Christopher Gale**

New Zealand

**Katie Gallacher**

UK

**James Gallagher**

Ireland

**Gisselle Gallego**

Australia

**Rebecca Ganann**

Canada

**David Gans**

USA

**Guodong Gao**

USA

**Jian Gao**

USA

**Marcial Garcia-Rojo**

Spain

**Karen Gardner**

Australia

**Allan Garland**

Canada

**Paul Garner**

UK

**James Gaughan**

UK

**Robin Gauld**

New Zealand

**Lara Gautier**

France

**Florian Gebreiter**

UK

**Alison Gee**

Australia

**Jo Geere**

UK

**Katrin Gehring**

Switzerland

**Alexander Geissler**

Germany

**Stacie Geller**

USA

**Becky Genberg**

USA

**Stephen Genuis**

Canada

**Pradeep Paul George**

Singapore

**Max Geraedts**

Germany

**Karen Gerard**

UK

**Saswata Ghosh**

India

**Swapnil Ghotane**

UK

**Francesco Gianfagna**

Italy

**Andry Giannakou**

Cyprus

**Maggie Gibson**

Canada

**Rod Gibson**

UK

**Julie Gilbert**

Canada

**Julia Gilmartin**

UK

**Sarah Gimbel**

USA

**Rosa Gini**

Italy

**Liane Ginsburg**

Canada

**Michael Gionfriddo**

USA

**Carlo Bruno Giorda**

Italy

**Edmond Girasek**

Hungary

**Stefan Gisin**

Switzerland

**Sophie Githinji**

Kenya

**Jody Hoffer Gittell**

USA

**Brian Godman**

Sweden

**Dianne Goeman**

Australia

**Katja Goetz**

Germany

**Lisa Gold**

Australia

**Rachel Gold**

USA

**Shaun Goldfinch**

Fiji

**David Goldsbury**

Australia

**Daniel Goldstein**

USA

**Marc Goldstein**

USA

**Sarah Gollust**

USA

**Barbara Gomes**

UK

**Francesc Xavier Gomez-Olive Casas**

South Africa

**Antonio Gómez-Outes**

Spain

**Eduardo Gonzalez-Pier**

Mexico

**María Cecilia González-Robledo**

Mexico

**Claire Goodman**

UK

**Catherine Goodman**

Kenya

**Yelena Gorina**

USA

**B L Gorissen**

Netherlands

**Holger Gothe**

Austria

**Lesley Gotlib Conn**

Canada

**Natasha Gous**

South Africa

**Pedro Gozalo**

USA

**Markus Grabka**

Germany

**Sandra Grace**

Australia

**Jonathan Graffy**

UK

**Chris Graham**

UK

**Gonzalo Grandes**

Spain

**Grazia Grazzini**

Italy

**Laurie Grealish**

Australia

**Esther Green**

Canada

**Dan Greenberg**

Israel

**William Greene**

USA

**David Greenfield**

Australia

**Trisha Greenhalgh**

UK

**Catherine Grenier**

France

**Peter Grimison**

Australia

**Karen Grimmer**

Australia

**Peter Groenewegen**

Netherlands

**Vigdis Abrahamsen Grøndahl**

Norway

**Charu Grover**

Australia

**Andrea Gruneir**

Canada

**Larry Gruppen**

USA

**Kristina Gryboski**

USA

**Enza Gucciardi**

Canada

**Mara Guerreiro**

UK

**P Cristian Gugiu**

USA

**Janice Gullick**

Australia

**Martin Gulliford**

UK

**How-Ran Guo**

Taiwan

**Indrani Gupta**

India

**Birgitta Å Gustafsson**

Sweden

**Lars L Gustafsson**

Sweden

**Inaki Gutierrez**

Spain

**Sally Guttmacher**

USA

**Astrid Guttmann**

Canada

**Sudesh Gyawali**

Nepal

**Hanna Gyllensten**

Sweden

**Yossi Hadad**

Israel

**Anna Häger Glenngård**

Sweden

**Anna Bettina Haidich**

Greece

**Peter Hajek**

UK

**Mark Hall**

USA

**Dagmar Haller**

Switzerland

**Pinchas Halpern**

Israel

**Christopher Halpin**

USA

**Kate Halton**

Australia

**Amy Halverson**

USA

**Syed Usman Hamdani**

Pakistan

**Robert Hamm**

USA

**Antje Hammer**

Germany

**David Hanauer**

USA

**Amresh Hanchate**

USA

**Nicola Hancock**

UK

**Rene Handschu**

Germany

**Johanna Hanefeld**

UK

**Breffni Hannon**

Canada

**Anne Helen Hansen**

Norway

**Bo Terning Hansen**

Norway

**Kristian Hansen**

UK

**Louise Hansen**

Denmark

**Claudia Hanson**

Sweden

**Piya Hanvoravongchai**

Thailand

**Yanhua Hao**

China

**Patrick Hardigan**

USA

**Jochen Hardt**

Germany

**Melanie Harling**

Germany

**Mark Harris**

Australia

**Mark Harrison**

UK

**Michael Harrison**

USA

**Simon Harrison**

Australia

**Christine Hartmann**

USA

**Abraham Hartzema**

USA

**Mohammed Harun-Or-Rashid**

Japan

**Carol Harvey**

Australia

**Gill Harvey**

UK

**M Hassanein**

UK

**Henna Hasson**

Sweden

**Arvid Steinar Haugen**

Norway

**Mona Haugum**

Norway

**Leslie Hausmann**

USA

**Rachel Havyer**

USA

**Nikki Hawkins**

USA

**Mark Hawley**

UK

**Alan Haycox**

UK

**Qusay Haydour**

USA

**Philip Haywood**

Australia

**Na He**

China

**Larry Hearld**

USA

**Marilynne Hebert**

Canada

**Oskari Heikinheimo**

Finland

**Michele Heisler**

USA

**Ramzi Helewa**

Canada

**Karla Hemesath**

USA

**Jane Henderson**

UK

**Michelle Hendriks**

Netherlands

**Kène Henkens**

Netherlands

**Peter Herbison**

New Zealand

**Abraham Herbst**

South Africa

**Pauline Heslop**

UK

**Gijs Hesselink**

Netherlands

**Suzanne Heurtin-Roberts**

USA

**Sarah Hewlett**

UK

**Sharon Hewner**

USA

**Peter Heywood**

Australia

**Judith Hibbard**

USA

**Irene J Higginson**

UK

**Sue Hignett**

UK

**Helmut Hildebrandt**

Germany

**Mickael Hiligsmann**

Netherlands

**Peter Hill**

Australia

**Janet Hiller**

Australia

**Susan Hillier**

Australia

**Peter Hindley**

UK

**David Hipgrave**

Australia

**Lisa Hirschhorn**

USA

**Karen Hirschman**

USA

**Jennifer Hirshfeld-Cytron**

USA

**Jane Hirst**

UK

**Helena Hnilicová**

Czech Republic

**Vivian Ho**

USA

**Matthias Hoben**

Canada

**Matthias Hochadel**

Germany

**Marilyn Hodgins**

Canada

**Erik Hoencamp**

Netherlands

**Alexander Hoerbst**

Austria

**Thomas Hoerger**

USA

**Aubri Hoffman**

USA

**Barbara Hoffmann**

Germany

**Tammy Hoffmann**

Australia

**Michael Höfler**

Germany

**Hans Högberg**

Sweden

**Hans V Hogerzeil**

Netherlands

**Hans Hogerzeil**

Switzerland

**Petra Högger**

Germany

**Christa Hojlo**

USA

**Monika Hollander**

Netherlands

**Kathleen Holloway**

Switzerland

**Lewis B. Holmes**

USA

**Caroline Homer**

Australia

**Jin Pyo Hong**

South Korea

**Danielle Hoover**

USA

**Ann Hopton**

UK

**Monjurul Hoque**

South Africa

**Frances Horgan**

Ireland

**Klaus Hornetz**

Germany

**Simon Horsburgh**

New Zealand

**Krishna Hort**

Australia

**Salih Hosoglu**

Turkey

**David Hotchkiss**

USA

**Kreshnik Hoti**

Australia

**Allan House**

UK

**Tami Howe**

New Zealand

**Susan Hrisos**

UK

**An-Fu Hsiao**

USA

**Hui-Min Hsieh**

Taiwan

**Ya-Seng Hsueh**

Australia

**Shanlian Hu**

China

**Yanping Huang**

Hong Kong

**Sean Shenghsiu Huang**

USA

**Kun Huang**

China

**Marco Huesch**

USA

**David Hui**

USA

**Robbert Huijsman**

Netherlands

**Mark Hull**

Canada

**Marjan Hummel**

Netherlands

**Debbie Humphries**

USA

**Benjamin Hunter**

UK

**Rachael Hunter**

UK

**Krista Huot**

USA

**Catherine Hurley**

Australia

**Samia Hurst**

Switzerland

**Raymond Hutubessy**

Switzerland

**Ellen Idler**

USA

**Francesca Ieva**

Italy

**Friedrich Ihler**

Germany

**Shunya Ikeda**

Japan

**David Ikkersheim**

Netherlands

**Zubairu Iliyasu**

Nigeria

**Yuichi Imanaka**

Japan

**Devon Indig**

Australia

**Tor Ingebrigtsen**

Norway

**Richard Ingram**

USA

**David Inwald**

UK

**Uma M Irfan**

Pakistan

**Tadashi Ishida**

Japan

**M. Mofizul Islam**

Australia

**Leyla Ismayilova**

USA

**Hiroto Ito**

Japan

**Kouta Ito**

USA

**Hilde Hestad Iversen**

Norway

**Valentina Cabral Iversen**

Norway

**Chimaraoke Izugbara**

Nigeria

**Christian Olaf Jacke**

Germany

**Claire Jackson**

Australia

**Debra Jackson**

South Africa

**Diana Jackson**

UK

**Susan Jaglal**

Canada

**Albrecht Jahn**

Germany

**Gro Jamtvedt**

Norway

**Monika Janda**

Australia

**Heidi Janssen**

Australia

**Lina Jaruseviciene**

Lithuania

**Sara Javanparast**

Australia

**Masood Jawaid**

Pakistan

**Anuradha Jayanti**

UK

**Anupam Jena**

USA

**Natasja Koitzsch Jensen**

Denmark

**Marija Jevtic**

Serbia

**Huanguang Jia**

USA

**Liu Jianxun**

China

**Masahito Jimbo**

USA

**Chenggang Jin**

China

**Huajie Jin**

UK

**Mark R D Johnson**

UK

**Peter Johnson**

USA

**Bridget Johnston**

UK

**Jukka Jokinen**

Finland

**Carys Jones**

UK

**Lorelei Jones**

UK

**Ray Jones**

UK

**Grace Joshy**

Australia

**Aleksandar Jovanovic**

Serbia

**Noel Juban**

Philippines

**Gyuchan Thomas Jun**

UK

**Ulrike Junius-Walker**

Germany

**Mary Justin-Temu**

Tanzania

**Haytham Kaafarani**

USA

**Axel Kaehne**

UK

**David Kaelber**

USA

**Natan Kahan**

Israel

**Ichiro Kai**

Japan

**Daphne Kaitelidou**

Greece

**Dan Kajungu**

Uganda

**László Kalabay**

Hungary

**Minal Kale**

USA

**Soumik Kalita**

India

**Lisa Kalman**

USA

**Lalit Kalra**

UK

**Eva Kaltenthaler**

UK

**Mary Kamb**

USA

**Taro Kamigaki**

Japan

**Abir Kanaan**

USA

**Donghun Kang**

South Korea

**Tipaporn Kanjanarach**

Thailand

**Hyacinthe Kankeu Tchewonpi**

France

**Lucy Kanya**

Kenya

**Dharmi Kapadia**

UK

**Anup Karan**

India

**Rajendra Karkee**

Nepal

**Jon Karnon**

Australia

**Monika Kastner**

Canada

**Gohei Kato**

Japan

**Noriko Kato**

Japan

**Mary Kawonga**

South Africa

**Kassoum Kayentao**

Mali

**Nicola Kayes**

New Zealand

**Peter Kazembe**

Malawi

**Benjamin Kearns**

UK

**Guy Kegels**

Belgium

**Margaret Kelaher**

Australia

**Anita Keller**

Switzerland

**Mary Lou Kelley**

Canada

**Anne Kennedy**

UK

**Patricia Kenny**

Australia

**Paula Kersten**

UK

**Stefan Kertesz**

USA

**Andrew Kestler**

Canada

**Jade Khalife**

Lebanon

**Jahangir Am Khan**

Bangladesh

**Pornsak Khortwong**

Thailand

**Juliet Kiguli**

Uganda

**Reinhold Kilian**

Germany

**Sue Kilpatrick**

Australia

**Sun-Young Kim**

USA

**James Kimani**

Kenya

**Shauna Kingsnorth**

Canada

**Russell Kirby**

USA

**Doris Kirigia**

Kenya

**Joses Muthuri Kirigia**

Congo

**Stephan Kirschner**

Germany

**Roman Kislov**

UK

**Alison Kitson**

Australia

**Suzanne Kiwanuka**

Uganda

**Lars Kjellin**

Sweden

**Egil Kjerstad**

Norway

**Caprice Knapp**

USA

**Herschel Knapp**

USA

**Martin Knapp**

UK

**Andrew Knighton**

USA

**Jennifer Knopp-Sihota**

Canada

**Naonori Kodate**

Ireland

**Leonardo Koeser**

UK

**Gerjo Kok**

Netherlands

**Maryse Kok**

Netherlands

**Michaela Kolbe**

Switzerland

**Daniela Koller**

Germany

**Masahide Kondo**

Japan

**Alice Kongsted**

Denmark

**Alexander Konnopka**

Germany

**Antonis Kontemeniotis**

Cyprus

**Evangelos Kontopantelis**

UK

**Sascha Köpke**

Germany

**Holly Korda**

USA

**Deborah Korenstein**

USA

**Thomas Kötter**

Germany

**Anita Kotwani**

India

**Camille Eric Kouam**

Canada

**Paul Kowal**

Switzerland

**Markus Kraus**

Austria

**Anand Krishnan**

India

**Solvejg Kristensen**

Denmark

**Troels Kristensen**

Denmark

**Jimmie Kristensson**

Sweden

**Kurt Kroenke**

USA

**Margaret Elizabeth Kruk**

USA

**Silvia Krumm**

Germany

**Elizabeth Krupinski**

USA

**Jeffrey Kullgren**

USA

**Chandan Kumar**

India

**B Nirmal Kumar**

UK

**Saravana Kumar**

Australia

**Vishwajeet Kumar**

India

**Abera Kumie**

Ethiopia

**Ludwig Kuntz**

Germany

**Cynthia Kurtz**

USA

**Talma Kushnir**

Israel

**Bethany Kwan**

USA

**Wang Kwua-Yun**

Taiwan

**Ilias-Ioannis Kyriopoulos**

Greece

**Ulrich Laaser**

Germany

**Tracey-Lea Laba**

Australia

**Laishram Ladusingh**

India

**Martine Lagace**

Canada

**Chandrakant Lahariya**

India

**Claas Lahmann**

Germany

**Maria Lahuerta**

USA

**Christiaan Lako**

Netherlands

**Héctor Lamadrid-Figueroa**

Mexico

**Stephen Lambert**

Australia

**Majda Lamkaddem**

Netherlands

**Bruce Landon**

USA

**Peter Lange**

Denmark

**Claudia Langebrake**

Germany

**Maaike Langelaan**

Netherlands

**Peter Larson**

USA

**Bruce Larson**

USA

**Francis Lau**

Canada

**Rosa Lau**

UK

**Michael Lauerer**

Germany

**Julie Lauffenburger**

USA

**Anthony Laverty**

UK

**Dana Lawrence**

USA

**Jeffrey Lazarus**

Denmark

**Amanda Leach**

Australia

**Jenine Leal**

Canada

**Andrew Chee Keng Lee**

UK

**Crystal Lee**

Australia

**Jae-Ho Lee**

South Korea

**Yu-Chen Lee**

UK

**Miaw-Chwen Lee**

Taiwan

**Mariska M G Leeflang**

Netherlands

**Jennifer Leeman**

USA

**Mengistu Legesse**

Ethiopia

**Sandra Leggat**

Australia

**David Legge**

Australia

**Beverly Leipert**

Canada

**Michael Leiter**

Canada

**Margus Lember**

Estonia

**Chryssoula Lemonidou**

Greece

**Kenneth L Leonard**

USA

**Aaron Leppin**

USA

**France Lert**

France

**Myles Leslie**

USA

**Ky Leung**

Hong Kong

**Ann Levin**

USA

**Carol Levin**

USA

**Robert Levine**

USA

**Terry Lewin**

Australia

**George Lewith**

UK

**Rui Li**

USA

**Gui Li**

China

**Ian Li**

Australia

**Guohong Li**

China

**Xiaohong Li**

China

**Yuyuan Li**

China

**Zhijian Li**

China

**Yi-Ran Li**

China

**Xiaoyan Li**

USA

**Ya-Hsin Li**

Taiwan

**Min Lian**

USA

**Ying Liang**

China

**Hsienming Lien**

Taiwan

**Margareta Lilja**

Sweden

**Rebbecca Lilley**

New Zealand

**Wen Kwang Lim**

Australia

**Teck-Onn Lim**

Malaysia

**Rosemary Lim**

UK

**Chulaporn Limwattananon**

Thailand

**Hualiang Lin**

China

**Chun-Pin Lin**

Taiwan

**Wender Lin**

Taiwan

**Wolfgang Linden**

Canada

**Helena Lindgren**

Sweden

**Tove Lindhardr**

Denmark

**Martin Lindstrom**

Sweden

**Matteo Lippi Bruni**

Italy

**Martin Lipsky**

USA

**George Liu**

Australia

**Hongjie Liu**

USA

**Hong Liu**

China

**Jinfen Liu**

China

**Songsong Liu**

UK

**Su Liu**

Hong Kong

**Xiaoyun Liu**

China

**Lucylynn Lizarondo**

Australia

**Peter Lloyd-Sherlock**

UK

**Carl Lombard**

South Africa

**Janet Long**

Australia

**Kim Longfield**

USA

**Yves Longtin**

Canada

**Luciane Cruz Lopes**

Brazil

**Fabiana Lorencatto**

UK

**Paula Lorgelly**

Australia

**Svetla Loukanova**

Germany

**Lesley Lowes**

UK

**Sam Lubner**

USA

**Tim Luckett**

Australia

**Wiebke Ludwig-Peitsch**

Germany

**Chi-Wai Lui**

Australia

**Jorge Luna**

USA

**Iréne Lund**

Sweden

**Sheng Luo**

USA

**May Nawal Lutfiyya**

USA

**Paula Luz**

Brazil

**Imogen Lyons**

UK

**Theodore Lystig**

USA

**Wei Ma**

China

**Thusitha Mabotuwana**

USA

**Christine Macarthur**

UK

**Marjorie Macdonald**

Canada

**Scott Macdonald**

Canada

**Anne Macfarlane**

Ireland

**Jane Macha**

Tanzania

**Adrian Mackenzie**

Canada

**Timothy Mackey**

USA

**Stuart Macleod**

Canada

**Malcolm Maclure**

Canada

**Deepak Rajaram Madi**

India

**Kenneth Maes**

USA

**Andreas Maetzel**

Canada

**Yodi Mahendradhata**

Indonesia

**Richard Mahoney**

South Korea

**Jenson Mak**

Australia

**Keijo Mäkelä**

Finland

**Matti Mäkelä**

Finland

**Madeeha Malik**

Pakistan

**Robert Malkin**

USA

**Margit Malmmose**

Denmark

**Mats Malqvist**

Sweden

**Stephen Maluka**

Tanzania

**Toshie Manabe**

Japan

**Rosemary Mander**

UK

**Lindsay Mangham-Jefferies**

UK

**Fatuma Manzi**

Tanzania

**Javier Mar**

Spain

**Bruno Marchal**

Belgium

**Giulio Marchesini**

Italy

**David Margel**

Israel

**Silvio Paolo Mariotti**

Switzerland

**Maureen Markle-Reid**

Canada

**Tania Markovic**

Australia

**Elsa Marques**

UK

**Paul Marschall**

Germany

**Andrea Marshall**

Australia

**Ernie Marshall**

UK

**Patrick Marshall**

UK

**Graham Martin**

UK

**Adriane Martin Hilber**

Switzerland

**Audibert Martine**

France

**Silvia Martinez Valverde**

Mexico

**Anne Martin-Matthews**

Canada

**Eliana Cristina Martins Miranda**

Brazil

**Michael Marx**

Germany

**Kijakazi Mashoto**

Tanzania

**Felix Masiye**

Zambia

**Caroline Masquillier**

Belgium

**Inke Mathauer**

Switzerland

**John D Mathews**

Australia

**Klazien Matter-Walstra**

Switzerland

**Stefano Mattioli**

Italy

**Marco Matucci-Cerinic**

Italy

**Massimo Maurici**

Italy

**Thandisizwe Redford Mavundla**

South Africa

**Carl May**

UK

**Serge Mayaka Ma-Nitu**

Belgium

**Jo Maybin**

UK

**Jose Mayordomo**

Spain

**Bongani Mawethu Mayosi**

South Africa

**Olena Mazurenko**

USA

**Pamela Mazzocato**

Sweden

**Patrick Mbindyo**

Kenya

**Nicholas Mc Innes**

Italy

**Kevin Mc Namara**

Australia

**Abigail Mcbirnie**

UK

**Gordon Mccord**

USA

**Ewan Mcdonald**

Australia

**Julie Mcdonald**

Australia

**Rosemary Mcginnes**

Australia

**Gerry Mcgivern**

UK

**Matthew Mcgrail**

Australia

**Cynthia Mcgrath**

USA

**Meredith Mcintyre**

Australia

**Suzanne Mckenzie**

Australia

**Andrew Mclachlan**

Australia

**Mary-Louise Mclaws**

Australia

**Susannah Mclean**

UK

**Christopher Mcleod**

Canada

**Monsey Mcleod**

UK

**Laurence Mcmahon**

USA

**Juliet Mcmullin**

USA

**Kelly Mcqueen**

USA

**Nicolas Meda Burkina**

Faso

**Lourdes Medina**

Puerto Rico

**Laurenz Meier**

Switzerland

**Sarah Meier**

USA

**Nadja Meisterhans**

Germany

**Cara Meixner**

USA

**Michelle Mello**

USA

**Shanthi Mendis**

Switzerland

**Matthew Menear**

Canada

**Qingyue Meng**

China

**Stephen Mennemeyer**

USA

**Emmanouil Mentzakis**

UK

**Nicolas Menzies**

USA

**Joseph Menzin**

USA

**Lisa Merry**

Canada

**Andrea Messori**

Italy

**Herman Meulemans**

Belgium

**Lisbet Meurling**

Sweden

**Thorsten Meyer**

Germany

**Gesine Meyer-Rath**

South Africa

**Bernhard Michalowsky**

Germany

**Kristien Michielsen**

Belgium

**Nicos Middleton**

Cyprus

**Evelinn Mikkelsen**

Netherlands

**Anneli Milen**

Finland

**Anton Miller**

Canada

**Edward Miller**

USA

**Raffi Miller**

USA

**Robert Miller**

USA

**Peggy Millson**

Canada

**Milan Milosevic**

Croatia

**William Min**

USA

**Mirella Minkman**

Netherlands

**Karin Minnie**

South Africa

**Silvano Mior**

Canada

**Jose Joaquin Mira**

Spain

**Nicholas Mitsakakis**

Canada

**Mitsunori Miyashita**

Japan

**Paul Mkandawire**

Canada

**Blandina Theophil Mmbaga**

Norway

**Rikke Moe**

Norway

**Deepika Mohan**

USA

**Katia Mohindra**

Canada

**David Mohr**

USA

**Mohammed Mohsin**

Australia

**Andrew Molodynski**

UK

**Shin-Ichi Momomura**

Japan

**Floriana Monciotti**

Italy

**Karen Monsen**

USA

**Ali Montazeri**

Iran

**Jesus Montero-Marin**

Spain

**Anthony Montgomery**

Greece

**Ann Moore**

USA

**David Moore**

Canada

**Jennifer Moore**

New Zealand

**Lynne Moore**

Canada

**Rachael Moorin**

Australia

**Mwifadhi Mrisho**

Tanzania

**Ashar Muhammad Malik**

Pakistan

**Axel Mühlbacher**

Germany

**Oscar J. Mujica**

USA

**Susmita Mukhopadhyay**

India

**Caroline Mulvaney**

UK

**Zubia Mumtaz**

Canada

**Kenneth Munge**

Kenya

**Fiona Munro**

UK

**Tariq Munshi**

Canada

**Mohammad Hassan Murad**

USA

**Adrianna Murphy**

UK

**Elizabeth Murray**

UK

**Jenni Murray**

UK

**Nandita Murukutla**

India

**Wilbroad Mutale**

Zambia

**David Muthaka**

Kenya

**Conrad Muzoora**

Uganda

**Corrie Myburgh**

Denmark

**Dries Myny**

Belgium

**Julie Mytton**

UK

**Juliet Nabyonga Orem**

Uganda

**Matthias Nachtnebel**

Australia

**Farooq Naeem**

Pakistan

**Kent Nakamoto**

USA

**Toshimi Nakanishi**

Japan

**Ireen Namakhoma**

Malawi

**Susan Nancarrow**

Australia

**Jagdeep Nanchahal**

UK

**Takashi Naruse**

Japan

**Rose Nathan**

Tanzania

**Corina Naughton**

UK

**Mwidimi Ndosi**

UK

**Sume Ndumbe-Eyoh**

Canada

**Graham Neilsen**

Australia

**Maria Ines B Nemes**

Brazil

**Sandeep S Nerkar**

Sweden

**Jonathan Neufeld**

USA

**Duncan Neuhauser**

USA

**Josh Neumiller**

USA

**William Newbrander**

USA

**Phung Anh Nguyen**

Taiwan

**Ha Nguyen**

Australia

**Thinh Nguyen**

Australia

**Pham Nguyen Bang**

Vietnam

**Michael B Nichol**

USA

**Paul Nicholson**

UK

**Nicola Nicolotti**

Italy

**Jason Xin Nie**

Canada

**Jens-Uwe Niehoff**

Germany

**Signe Smith Nielsen**

Denmark

**Barnabas Njozing**

Kenya

**Edward Nketiah-Amponsah**

Ghana

**Godswill Nnaji**

Nigeria

**Lucia Nobilio**

Italy

**Natasha Noble**

Australia

**Haruko Noguchi**

Japan

**Susan L Norris**

USA

**Shane Norris**

South Africa

**Nicola North**

New Zealand

**Dino Numerato**

UK

**Roberto Nuño-Solinís**

Spain

**Robin Nwankwo**

USA

**Francis Obare**

Kenya

**Jon Oyvind Odland**

Norway

**Brian Odonoghue**

Australia

**Olumuyiwa Odusanya**

Nigeria

**Pia Oedewald**

Finland

**Ju Lee Oei**

Australia

**Uzor Ogbu**

USA

**Russel Ogden**

Canada

**Tinuade Ogunlesi**

Nigeria

**In-Hwan Oh**

South Korea

**Jude Uzoma Ohaeri**

Nigeria

**Bernadette O’Hare**

Malawi

**Elialilia S Okello**

Uganda

**Christian Okolo**

Nigeria

**Oladepo Oladimeji**

Nigeria

**Joyce Olenja**

Kenya

**John Oliffe**

Canada

**Juan Oliva-Moreno**

Spain

**Kathryn Oliver**

UK

**Elizabeth Oloruntoba**

Nigeria

**Samuel Olowookere**

Nigeria

**Lars-Eric Olsson**

Sweden

**Femi Omole**

South Africa

**Joseph Onakewhor**

Nigeria

**Ciaran O’Neill**

Ireland

**Jason Ong**

Australia

**Pierre Ongolo-Zogo**

Cameroon

**Charles Opondo**

Kenya

**Dermot O’Reilly**

UK

**Aaron Orkin**

Canada

**Carljohan Orre**

Sweden

**Jacques Orvain**

France

**Dominik Ose**

Germany

**Helmut Ostermann**

Germany

**Herwig Ostermann**

Austria

**Laysha Ostrow**

USA

**Ronan O’Sullivan**

Ireland

**Martin Ota**

Congo

**Angel Otero**

Spain

**Wilma Otten**

Netherlands

**Lixin Ou**

Australia

**Jan Oyebode**

UK

**Doruk Ozgediz**

USA

**Jamie Padmore**

USA

**Andrew Page**

Australia

**Kathleen Page**

USA

**Charles John Palenik**

USA

**Luigi Palestini**

Italy

**Lawrence Palinkas**

USA

**Laura Palombi**

USA

**Pradeep Panda**

India

**Massimiliano Panella**

Italy

**Supasit Pannarunothai**

Thailand

**George Papachristou**

Greece

**Evelina Pappa**

Greece

**Hong-Jae Park**

New Zealand

**Young Sik Park**

South Korea

**Sophie Park**

UK

**Louise Parker**

USA

**Vicki Parker**

Australia

**Aimee Parnell**

USA

**Adriana Parrella**

Australia

**David Parry**

New Zealand

**Gareth Parry**

USA

**Matthew Parsons**

New Zealand

**Helen Parsons**

USA

**Minal Patel**

USA

**Preeti Patel**

UK

**Andrea Patey**

Canada

**Elisabetta Patorno**

USA

**Sue Pavitt**

UK

**Ioanna Pavlopoulou**

Greece

**Milena Pavlova**

Netherlands

**Manjiri Pawaskar**

USA

**Christopher Pearce**

Australia

**Rupert Pearse**

UK

**Nancye May Peel**

Australia

**Jose Peeters**

Netherlands

**Salvador Peiró**

Spain

**Ashleigh Pell**

UK

**James Peltier**

USA

**Mark Pennington**

UK

**Beata Peplonska**

Poland

**Jose Pereira**

Canada

**Ricardo Perez-Cuevas**

Mexico

**Virtudes Perez-Jover**

Spain

**David Perkins**

Australia

**John Perry**

Canada

**Stephen Persell**

USA

**Gregory Peterson**

Australia

**Stefan Peterson**

Sweden

**Anastas Philalithis**

Greece

**Mit Philips**

Belgium

**Ceri Phillips**

UK

**James Phillips**

USA

**Lawrence Phillips**

UK

**Lyn Phillipson**

Australia

**Fiammetta Piersigilli**

Italy

**Arwen Pieterse**

Netherlands

**Clément Pimouguet**

France

**Hilary Pinnock**

UK

**Aarão Mendes Pinto Neto**

Brazil

**Jorge Pinzon**

Canada

**Stephen Pitts**

USA

**Anne Marie Plass**

Netherlands

**Christian Plessen**

Denmark

**Thomas Plochg**

Netherlands

**Ronald Plotnikoff**

Australia

**Rhys Pockett**

UK

**Dan Poenaru**

Canada

**Subhash Pokhrel**

UK

**Jeannette Pols**

Netherlands

**Marie-Pascale Pomey**

Canada

**Catherine Pope**

UK

**Brenten Popiel**

USA

**Aron Frederik Popov**

Germany

**Andrea Poscia**

Italy

**Douwe Postmus**

Netherlands

**Otto Melchior Poulsen**

Denmark

**Koen B. Pouwels**

Netherlands

**Colin Powell**

UK

**Timothy Powell-Jackson**

UK

**Alexis Pozen**

USA

**Wasana Prasitsuebsai**

Thailand

**Devarsetty Praveen**

India

**George Praygod**

Tanzania

**Dawn Prentice**

Canada

**Panagiotis Prezerakos**

Greece

**Mirela Prgomet**

Australia

**Morgan Price**

Canada

**Kathleen Pritchard**

Canada

**Ari Probandari**

Indonesia

**Birgit Prodinger**

Switzerland

**Ana Progovac**

USA

**Supannee Promthet**

Thailand

**Wendy Prudhomme O’Meara**

Kenya

**Dimitri Prybylski**

USA

**Jacqueline Pugh**

USA

**Sue Pullon**

New Zealand

**Rebecca Purc-Stephenson**

Canada

**Sampsa Puttonen**

Finland

**Peter Pype**

Belgium

**Feng Qian**

USA

**Xuezheng Qin**

China

**Troy Quast**

USA

**Wilm Quentin**

Germany

**Sally Quilligan**

UK

**Julie Quinlivan**

Australia

**Atonu Rabbani**

Bangladesh

**Fauziah Rabbani**

Pakistan

**Giovanni Rabito**

Switzerland

**Beth Rachlis**

Canada

**Rajiv Radhakrishnan**

USA

**Alberto Raggi**

Italy

**Lisa Rahangdale**

USA

**Anna Rahman**

USA

**Peter Raich**

USA

**Risto Raivio**

Finland

**Vahid Rakhshan**

Iran

**Mark Ralston**

USA

**Bram Ramaekers**

Netherlands

**Peter Rambau**

Tanzania

**Edmond Ramly**

USA

**Sue Randall**

Australia

**Gurprit Randhawa**

Canada

**Erik Ranschaert**

Belgium

**Jonas Ranstam**

Sweden

**Tapio Rantanen**

Finland

**Lars Rasmussen**

Denmark

**Freya Rasschaert**

Belgium

**Nithima Ratanasit**

Thailand

**Mihi Ratima**

New Zealand

**Ramin Ravangard**

Iraq

**John Read**

UK

**Bernd Rechel**

UK

**Siddharta Reddy**

USA

**J A Reekers**

Netherlands

**Jemma Regan**

UK

**Krishna Regmi**

UK

**Jaroslaw Regula**

Poland

**Colin Reid**

Canada

**Stephen Reid**

South Africa

**Stewart Reid**

Zambia

**Thomas Reinhold**

Germany

**William Renwick**

Australia

**James Reschovsky**

USA

**Serge Resnikoff**

Switzerland

**Joseph Restuccia**

USA

**Kelly Reveles**

USA

**Debra Revere**

USA

**Heidi Reynolds**

USA

**Joanna Reynolds**

UK

**Abbas Rezaianzadeh**

Iran

**Reza Rezayatmand**

Netherlands

**Todd Rice**

USA

**Rebecca Richards-Kortum**

USA

**Solina Richter**

Canada

**Wietske Rienstra**

Netherlands

**Michael Rigby**

Ireland

**Ian Ring**

Australia

**Marit By Rise**

Norway

**Gilbert Ritschard**

Switzerland

**Kerstin Roback**

Sweden

**Adam Roberts**

UK

**Drucilla Roberts**

USA

**Jackie Robinson**

New Zealand

**Jaime Robledo**

Colombia

**Monica Robotin**

Australia

**Dylan Roby**

USA

**Peter Rockers**

USA

**Rosa Rodriguez-Monguio**

USA

**Cecilie Roe**

Norway

**Morwenna Rogers**

UK

**James Rohrer**

USA

**Martin Roland**

UK

**Sarah Rominski**

USA

**Janetta Roos**

South Africa

**Susan Rose**

USA

**Laura Rosella**

Canada

**Michael Rosen**

USA

**Pauline Rosenau**

USA

**Tom Ross**

USA

**Andrea Rotolo**

Italy

**Ronen Rozenblum**

USA

**Juan Ruano**

Spain

**Leonard Rubenstein**

USA

**Daniel Rubin**

USA

**Maria Rubio-Valera**

Spain

**Gavin Rudge**

UK

**Robert Rudin**

USA

**Claudia Rudroff**

Germany

**Peter Ruesch**

Switzerland

**Myriam Ruiz-Rodriguez**

Colombia

**Giuliano Russo**

Portugal

**Elizeus Rutebemberwa**

Uganda

**Geert Rutten**

Netherlands

**Joanne Ryan**

Australia

**Carla Sabariego**

Germany

**Lindsay Sabik**

USA

**Mohsen Sadatsafavi**

Canada

**Debasish Saha**

Belgium

**Olivier Saint-Lary**

France

**Adnan Saithna**

UK

**Priyanka Saksena**

Switzerland

**Rehana Salam**

Pakistan

**Abdus Salam**

Malaysia

**Ellinor Salander Renberg**

Sweden

**Guillermo Salinas-Escudero**

Mexico

**Miguel Salinero-Fort**

Spain

**Andrew Salner**

USA

**Brendan Saloner**

USA

**Stacie Salsbury**

USA

**Katherine Salter**

Canada

**Domenico Salvatore**

Italy

**Jane Sandall**

UK

**Magnus Sandberg**

Sweden

**Uwe Sander**

Germany

**Lars Sandman**

Sweden

**Felipe Sandoval**

Japan

**Gabriel Sanfélix-Gimeno**

Spain

**Claudia Sanmartin**

Canada

**Milena Sant**

Italy

**Malay Sarkar**

India

**Malabika Sarker**

Bangladesh

**Faruk Sarkinfada**

Nigeria

**Marco Sartirana**

Italy

**Sumathi Sathya**

India

**Azusa Sato**

UK

**Mikiya Sato**

Japan

**Stefan Sauerland**

Germany

**Saw Saw Saw**

Myanmar

**Sylvia Sax**

Germany

**Shane Scahill**

New Zealand

**Sasha Scambler**

UK

**Marco Scarpa**

Italy

**Frederieke Schaafsma**

Australia

**Juergen Schaefer**

Germany

**Francois Schellevis**

Netherlands

**Jeffrey Scherrer**

USA

**Virginia Schmied**

Australia

**Helen Schneider**

South Africa

**Michael Schneider**

USA

**Veronika Schoeb**

Switzerland

**Isabelle Scholl**

Germany

**Christoph Scholz**

Germany

**Miranda Schram**

Netherlands

**Jonas Schreyögg**

Germany

**Paul Schultz**

USA

**Richard Schulz**

USA

**Nadine Schuurman**

Canada

**Hilary Schwandt**

USA

**Rene Schwendimann**

Switzerland

**David Scrimgeour**

Australia

**David Seder**

USA

**Laurence Seematter-Bagnoud**

Switzerland

**Joel Segel**

USA

**Hanna Seidling**

Germany

**Polona Selic**

Slovenia

**Sakthivel Selvaraj**

India

**Salaam Semaan**

USA

**Catherine Sermet**

France

**Walter Sermeus**

Belgium

**Tanya Seshadri**

India

**Elizabeth Seston**

UK

**Nick Sevdalis**

UK

**Melchior Seyfarth**

Germany

**Omid Shabestari**

Canada

**Baiju Shah**

Canada

**Madhusudana Shampur**

India

**Vandad Sharifi**

Iran

**Urvashi Sharma**

UK

**Adam Sharp**

USA

**Alison Shaw**

UK

**Charles Shaw**

UK

**Dorothy Shaw**

Canada

**L. Aubree Shay**

USA

**Ian Sheerin**

New Zealand

**Paul Shekelle**

USA

**Jay Shen**

USA

**Bryan Shepherd**

USA

**Yuyan Shi**

USA

**Shu-Fang Shih**

Taiwan

**Chih-Yuan Shih**

Taiwan

**Sophy Shih**

Australia

**Nathan Shippee**

USA

**Amir Shmueli**

Israel

**N Shorbaji**

Switzerland

**Camille Short**

Australia

**Zhou Shoujun**

China

**Mohsin Sidat**

Mozambique

**Grigory Sidorenkov**

Netherlands

**Mark Siedner**

USA

**Rüdiger Siekmeier**

Germany

**Volkert Siersma**

Denmark

**Jacques Silber**

Israel

**Jonathan Silcock**

UK

**Martha Silva**

New Zealand

**Leickness Chisamu Simbayi**

South Africa

**Douglas Simkiss**

UK

**Edina Sinanovic**

South Africa

**Timo Sinervo**

Finland

**Daniel Singer**

USA

**Sara Singer**

USA

**Simone Singh**

USA

**Dan Siskind**

Australia

**Eric Siskind**

USA

**Priyadharshini Sivakumaran**

Australia

**Judith Sixsmith**

UK

**Steve Sizmur**

UK

**Ingeborg Strømseng Sjetne**

Norway

**Chris Skedgel**

Canada

**Kjersti Eeg Skudal**

Norway

**Susan Slade**

Australia

**Susan Slaughter**

Canada

**Nancy L Sloan**

USA

**Neil Smart**

Australia

**Cees Th. Smit Sibinga**

Netherlands

**Alison Smith**

UK

**Anthony Smith**

Australia

**Dean Smith**

USA

**Susan Smith**

Ireland

**Carolyn Smith-Morris**

USA

**Liz Smythe**

New Zealand

**Helene L Soberg**

Norway

**Paulina Sockolow**

USA

**Girish M Sogi**

India

**Mohammad Reza Sohrabi**

Iran

**Roy L. Soiza**

UK

**Maria Giuliana Solinas**

Italy

**Dominique Somme**

France

**David Sommerfeld**

USA

**Benjamin Sommers**

USA

**Mark Sonderup**

South Africa

**Hummy Song**

USA

**Yunjie Song**

USA

**Werner Soors**

Belgium

**Elizaveta Sopina**

Denmark

**Jacob Thorsted Sorensen**

Denmark

**Jan Sorensen**

Denmark

**Atsushi Sorita**

USA

**Sandra G. Sosa-Rubi**

Mexico

**Kyriakos Souliotis**

Greece

**Paulo Sousa**

Portugal

**Mikle South**

USA

**Alex Sox-Harris**

USA

**Olaitan Soyannwo**

Nigeria

**Adedoyin Soyibo**

Nigeria

**Donat Rudolf Spahn**

Switzerland

**Lene Spanager**

Denmark

**Stuart Speedie**

USA

**Neil Spicer**

UK

**Mark Spigt**

Netherlands

**Anne Spinewine**

Belgium

**Andrew Springer**

USA

**Jayadevan Sreedharan**

United Arab Emirates

**Shalini Sri Ranganathan**

Sri Lanka

**Anurag Srivastava**

India

**Jeong Seok Sro**

South Korea

**Catherine St. Hill**

USA

**Cecilia Stålsby Lundborg**

Sweden

**Baudouin Standaert**

Belgium

**Hilary Standing**

UK

**Victoria Stanhope**

USA

**Susan Stapleton**

USA

**Laura Steinhardt**

USA

**Gregory Stevens**

USA

**Joanna Stewart**

New Zealand

**Jane Stewart**

UK

**Alessandro Stievano**

Italy

**Karyn Stitzenberg**

USA

**Jill Stocks**

UK

**Andrew Stoddart**

UK

**Johannes Stoelwinder**

Australia

**Emma Stokes**

Ireland

**Matthias Stoll**

Germany

**Jamie Stone**

USA

**Marianne Storm**

Norway

**Jackie Street**

Australia

**Matej Stuhec**

Slovenia

**Prasanth Subrahmanian**

India

**Dawn Sugarman**

USA

**Takehiro Sugiyama**

Japan

**Sarah Sullivan**

UK

**Sheena Sullivan**

Australia

**Elinor Sullivan**

USA

**Qi Sun**

China

**Reijo Sund**

Finland

**Gerdt Sundstrom**

Sweden

**Wayne Sunman**

UK

**Rosa Sunol**

Spain

**Tarja Suominen**

Finland

**Paibul Suriyawongpaisal**

Thailand

**György Surján**

Hungary

**Igor Svab**

Slovenia

**Elisabeth Svensson**

Sweden

**Dace Svikis**

USA

**Michael Swanoski**

USA

**Deborah Swinglehurst**

UK

**Shabbir Syed-Abdul**

Taiwan

**Miriam Taegtmeyer**

UK

**Saeed Taheri**

Iran

**Hideto Takahashi**

Japan

**Hiromi Takahashi-Omoe**

Japan

**Zenobia Talati**

Australia

**Giorgio Tamburlini**

Italy

**Joseph Tan**

Canada

**Siok Swan Tan**

Netherlands

**Ryan Tandjung**

Switzerland

**Min Moon Tang**

Malaysia

**Shenglan Tang**

USA

**Wei Tang**

Japan

**Sripen Tantivess**

Thailand

**Tawesak Tanwandee**

Thailand

**Sihai Tao**

China

**Elizabeth Tarlov**

USA

**Sandra Tarquinio**

Brazil

**Robert Tate**

Canada

**Paula Tavrow**

USA

**Katja Taxis**

Netherlands

**Chelsea Taylor**

Australia

**Judy Taylor**

Australia

**Nicholas Taylor**

Australia

**Natalie Taylor**

Australia

**Sandra Taylor**

USA

**Katherine Taylor Haynes**

USA

**Gary Teare**

Canada

**Cristian Tebé**

Spain

**Marleen Temmerman**

Belgium

**Martha Ann Terry**

USA

**Angela Testi**

Italy

**Dilip Thandassery**

India

**Gudrun Thengisdottir**

UK

**Horst Thiermann**

Germany

**Jill Thistlethwaite**

Australia

**Aliki Thomas**

Canada

**Anne Thomas**

USA

**Bejoy Thomas**

Canada

**Roanne Thomas**

Canada

**Sandra Thompson**

Australia

**Ann Thomson**

UK

**William Murray Thomson**

New Zealand

**Richard Thomson**

UK

**Kevan Thorley**

UK

**Kamala Thriemer**

Australia

**Colin Thunhurst**

UK

**Lau Thygesen**

Denmark

**Stephanie Tierney**

UK

**Maike Tietschert**

Netherlands

**Stephen Timmons**

UK

**Samuel Tisherman**

USA

**Hale Toklu**

USA

**Krzysztof Tomaszewski**

Poland

**Sara Tomczyk**

Ethiopia

**Toshiyoshi Tominaga**

Japan

**Stephanie Topp**

Zambia

**Kwasi Torpey**

Nigeria

**Hidenori Toyoda**

Japan

**Valeria D. Tozzi**

Italy

**Joan Tranmer**

Canada

**Gerd Tranø**

Norway

**Jeanette Trauth**

USA

**Andrea Tricco**

Canada

**Ursula Trummer**

Austria

**Jeroen Trybou**

Belgium

**Yafang Tsai**

Taiwan

**Jack Tsai**

USA

**Feng-Jen Tsai**

Taiwan

**Girmay Tsegay**

Ethiopia

**Apostolos Tsiachristas**

Netherlands

**Miyuki Tsuchihashi-Makaya**

Japan

**Hong Anh Tu**

Netherlands

**Carole Tucker**

USA

**Lars Tummers**

Netherlands

**Janet Turan**

USA

**Joanne Turnbull**

UK

**Janette Turner**

UK

**Sheila Turner**

UK

**Ingrid Tyler**

Canada

**Kerry Uebel**

South Africa

**Maduka Ughasoro**

Nigeria

**Nkolika Uguru**

Nigeria

**Jennifer Uhrig**

USA

**Waqar Ulhassan**

Sweden

**Antje Ullrich**

Germany

**Jane Umeano-Enemuoh**

Nigeria

**Hiroyuki Umegaki**

Japan

**Rosa M. Urbanos-Garrido**

Spain

**Christine Urquhart**

UK

**Robin Urquhart**

Canada

**Maritta Valimaki**

Finland

**Mentore Vaccari**

Italy

**Anil Vaidya**

UK

**Vivian Valdmanis**

USA

**Luke Vale**

UK

**Janet Van Cleave**

USA

**Kate Van De Vooren**

Netherlands

**Elske Van Den Akker**

Netherlands

**Patricia Van Den Bemt**

Netherlands

**Neeltje Van Den Berg**

Germany

**Hendrik Van Den Bussche**

Germany

**Jan Van Der Meulen**

UK

**Ineke Van Der Wulp**

Netherlands

**Willem Herbert Van Harten**

Netherlands

**Richard Van Kleef**

Netherlands

**Josefien Van Olmen**

Belgium

**Catharina Van Oostveen**

Netherlands

**Joost Van Veen**

UK

**Carol Vandeusen Lukas**

USA

**Per Olav Vandvik**

Norway

**Kris Vanhaecht**

Belgium

**Özalp Vayvay**

Turkey

**Nerina Vecchio**

Australia

**Alicia Vedio**

UK

**Pepijn Vemer**

Netherlands

**Bruno Ventelou**

France

**Rafael Vercelino**

Brazil

**R.A. Verheij**

Netherlands

**Arnoud Verhoeff**

Netherlands

**Marcia Vervloet**

Netherlands

**Reinhold Vieth**

Canada

**Stefano Villa**

Italy

**Deborah Vincent**

USA

**Katherine Virgo**

USA

**Susan Vlack**

Australia

**Lorenz Von Seidlein**

Thailand

**Thomas Vreugdenburg**

Australia

**Bert Vrijhoef**

Singapore

**Victoria Anne Wade**

Australia

**Norman Waitzman**

USA

**Annika Waldmann**

Germany

**Agnes Walker**

Australia

**Kara Walker**

USA

**Amy Walker**

USA

**Emily Walkom**

Australia

**Emma Wallace**

Ireland

**John Walley**

UK

**Lars Wallin**

Sweden

**Bronagh Walsh**

UK

**Gill Walt**

UK

**Ulla Walter**

Germany

**Darren Walters**

Australia

**Stephen John Walters**

UK

**Edward Waltz**

USA

**Richard Wamai**

USA

**Nick Wan**

USA

**Anne Wand**

Australia

**Fahui Wang**

USA

**Guijing Wang**

USA

**Hufeng Wang**

China

**Hongmei Wang**

China

**Zhen Wang**

USA

**Jian Wang**

China

**Qing Wang**

China

**Ying Wang**

China

**Wei Wang**

Australia

**Christine Wann-Hansson**

Sweden

**Rhoda Wanyenze**

Uganda

**Jane Wardle**

UK

**Douglas Fraser Wares**

Switzerland

**Justin Waring**

UK

**Margaretha Warlé-Van Herwaarden**

Netherlands

**Charlotte Warren**

Kenya

**Narelle Warren**

Australia

**Steven Wartman**

USA

**Elizabeth Wasilevich**

USA

**Patrick Waterson**

UK

**Francois Watson**

South Africa

**Verity Watson**

UK

**Robert Wears**

USA

**William Weeks**

USA

**Li Wei**

UK

**Ji-Fu Wei**

China

**Xiaolin Wei**

China

**Matthias Weigl**

Germany

**John Wells**

Ireland

**Kristen Wells**

USA

**Susan Wells**

New Zealand

**Annalena Welp**

Switzerland

**Claus Wendt**

Germany

**Rhay-Hung Weng**

Taiwan

**Sophie Werkö**

Sweden

**Erik L. Werner**

Norway

**Colin West**

USA

**Laura Wherry**

USA

**Dean Whitehead**

New Zealand

**Stuart Whittaker**

South Africa

**David Whyatt**

Australia

**Siri Wiig**

Norway

**Heinrike Wilkens**

Germany

**Barbara Willey**

UK

**Anna Williams**

Australia

**Ann Williams**

USA

**Mark Williams**

USA

**Chad Williamson**

USA

**Sarah Willis**

UK

**Douglas Wilson**

South Africa

**Khin Than Win**

Australia

**Haije Wind**

Netherlands

**Lori Wingate**

USA

**Brad Winters**

USA

**Meg Wise**

USA

**Jens Witsch**

Germany

**Barbara Wizner**

Poland

**Eileen Wllis**

Australia

**Priscilla Wobil**

Ghana

**Leslie Wolf**

USA

**Carlos King Ho Wong**

Hong Kong

**Edwin Wong**

USA

**Geoff Wong**

UK

**Sabrina Wong**

Canada

**Rachael Wood**

UK

**Fiona Wood**

UK

**Richard Woodman**

Australia

**Cynthia Woodsong**

South Africa

**Edwin Wouters**

Belgium

**Frances Wright**

Canada

**Bei Wu**

USA

**Lesley Wye**

UK

**Kaspar Wyss**

Switzerland

**Anping Xie**

USA

**Jian-Xiang Xie**

China

**Lingzhong Xu**

China

**Ji-Jiang Yang**

China

**Li-Ye Yang**

China

**Yi-Hsin Yang**

Taiwan

**Philip Yanos**

USA

**Lorraine Yap**

Australia

**Marie Yap**

Australia

**Bobbi Jo Yarborough**

USA

**Aaron Yarlas**

USA

**Annalee Yassi**

Canada

**Barbara Yawn**

USA

**Bo Ye**

UK

**Xiaohua Ye**

China

**Wen Ye**

USA

**Peter Yellowlees**

Australia

**Laurann Yen**

Australia

**Hongmei Yi**

China

**Cheng-Har Yip**

Malaysia

**Jennifer Yip**

UK

**Candice Yong**

USA

**Hong Il Yoo**

UK

**Illhoi Yoo**

USA

**Jean Yoon**

USA

**Joyce You**

Hong Kong

**Tracey Young**

UK

**Sitaporn Youngkong**

Thailand

**Mustafa Younis**

USA

**Junhua Yu**

USA

**Beibei Yuan**

Sweden

**Zhaokang Yuan**

China

**Manwai Yuen**

Hong Kong

**Nicole Yurgin**

USA

**Mahmoud Zakherah**

Egypt

**Paola Zappa**

Switzerland

**Ehsan Zarei**

Iran

**Ruth Zaslansky**

Germany

**John Zeber**

USA

**Sten Zelle**

Netherlands

**Jennifer Zelmer**

Canada

**Wu Zeng**

USA

**Floriana Zennaro**

Italy

**Isik Zeytinoglu**

Canada

**Xinping Zhang**

China

**Zhitao Zhang**

China

**Jingping Zhao**

China

**Kai Zheng**

USA

**Lei Zhou**

China

**Liang Zhou**

China

**Quan Zhou**

China

**Carolyn Zhu**

USA

**Junya Zhu**

USA

**Joseph Zickafoose**

USA

**Maria Zolfo**

Belgium

**Diana Zuckerman**

USA

**Yvonne Zurynski**

Australia

